# Poly(δ-valerolactone)/Poly(ethylene-co-vinylalcohol)/β-Tricalcium Phosphate Composite as Scaffolds: Preparation, Properties, and In Vitro Amoxicillin Release

**DOI:** 10.3390/polym13010046

**Published:** 2020-12-24

**Authors:** Mohammed Badwelan, Mohammed Alkindi, Osama Alghamdi, Waseem Sharaf Saeed, Abdel-Basit Al-Odayni, Ali Alrahlah, Taieb Aouak

**Affiliations:** 1 Department of Oral and Maxillofacial Surgery, College of Dentistry, King Saud University, Riyadh 11545, Saudi Arabia; mnbadwelan@ksu.edu.sa (M.B.); malkindi@ksu.edu.sa (M.A.); oghamdi@ksu.edu.sa (O.A.); 2 Engineer Abdullah Bugshan Research Chair for Dental and Oral Rehabilitation, College of Dentistry, King Saud University, Riyadh 11545, Saudi Arabia; aalodayni@ksu.edu.sa (A.-B.A.-O.); aalrahlah@ksu.edu.sa (A.A.); 3 Restorative Dental Sciences Department, College of Dentistry, King Saud University, Riyadh 11545, Saudi Arabia; 4 Chemistry Department, College of Science, King Saud University, Riyadh 11451, Saudi Arabia

**Keywords:** poly(δ-valerolactone)/poly(ethylene-co-vinylalcohol)/β-tricalcium phosphate composite, physicochemical properties, pore interconnection, scaffold, mechanical properties, cell viability, amoxicillin dynamic release

## Abstract

Two poly(δ-valerolactone)/poly(ethylene-co-vinylalcohol)/β-tricalcium phosphate (PEVAL/PDVAL/β-TCP) composites containing an equal ratio of polymer and filled with 50 and 70 wt% of β-TCP microparticles were prepared by the solvent casting method. Interconnected pores were realized using the salt leached technique, and the porosity of the resulted composites was evaluated by the scanning electron microscopy (SEM) method. The homogeneity of the hybrid materials was investigated by differential scanning calorimetry (DSC) and X-ray diffraction (XRD) analysis. The prepared materials’ SEM images showed interconnected micropores that respond to the conditions required to allow their uses as scaffolds. The porosity of each scaffold was determined from micro computed tomography (micro-CT) data, and the analysis of the mechanical properties of the prepared materials was studied through the stress-strain compressive test. The proliferation test results used human mesenchymal stem cells (MSCs) to grow and proliferate on the different types of prepared materials, reflecting that the hybrid materials were non-toxic and could be biologically acceptable scaffolds. The antibacterial activity test revealed that incorporation of amoxicillin in the specimens could inhibit the bacterial growth of *S. aureus.* The in vitro study of the release of amoxicillin from the PEVAL/PDVAL/amoxicillin and PEVAL/PDVAL/β-TCP/amoxicillin drug carrier systems in pH media 7.4, during eight days, gave promising results, and the antibiotic diffusion in these scaffolds obeys the Fickian model.

## 1. Introduction

In the field of orthopedic and craniofacial surgery, bone tissue regeneration is a challenging procedure. Critical size bone defects that result from trauma, pathology, and fracture cause a significant clinical problem [1]. These bony defects require a bone graft, or its substitute, with osteoinductive or osteoconductive properties. Therapeutic approaches attempt the natural process of osseous regeneration by providing materials acting as scaffolding matrices, which maintain cellular attachment, migration, and proliferation to repair the bone defects [2]. Different natural polymeric materials, including starch, alginate, cellulose, silk, polysaccharides, gelatin, and collagen are extensively examined in tissue engineering due to their comparable physicochemical properties in the extracellular matrix of living tissue [3,4]. Therefore, only a very small number of these polymers have been selected for application as carriers in drug carrier systems and scaffolding preparation [5]. Despite these biological advantages cited, in particular biodegradability and biocompatibility, certain disadvantages, such as their poor mechanical properties, possible disease transmission, fast resorption rate, the possibility of causing an allergic reaction, rapid bone regeneration at initial stages, bone formation after implanting these matrices occurs over a long period, rapid degradation in vivo, difficultly maintaining structural integrity, and high cost due to its scant availability [6,7,8,9], are at the origin of the limitation of their large-scale application in the biomedical field.

To develop a material combining the characteristics necessary to be an adequate candidate in medical applications, in which a single product generally does not have them, different researchers have resorted to involving polymers associated with other compounds as hybrid materials by blending polymers or mixing polymers with fillers to solve various complications encountered in biomedical applications, in particular, bone repair. Indeed, for example, Kim and Joe [10] investigated three-dimensional (3D) blend scaffolds combining poly-caprolactone (PCL) with poly-lactic-co-glycolic acid (PLGA) using a multi-head deposition system technique for the regeneration of damaged tissues of organs in scaffold-based tissue engineering. The pores distribution in the scaffold prepared were uniform, size optimized, and pores interconnected. The compressive strength and modulus of the prepared scaffold were also adequate and were enough to maintain the scaffold’s architecture during in vitro cell experiments. Here, the PCL’s main disadvantage when used as scaffolds is its strongly hydrophobic nature, leading to non-specific protein adsorption [11,12,13]. Palumbo et al. [14] prepared a cyclo(Arg-Gly-Asp-D-Phe-Cys) (RGDC) functionalized hybrid biomaterial starting from poly(l-lactic acid) (PLLA) and an amorphous α,β-poly(N-2-hydroxyethyl) (2-aminoethylcarbamate)-d,l-aspartamide-graft-polylactic acid (PHEA-EDA-*g*-PLA) copolymer to achieve more efficient control of osteoblast adhesion and diffusion on the three-dimensional (3D) scaffolds. Cell culture test carried out on preosteoblastic cells (MC3T3-E1) incubated with scaffolds has evidence of cell adhesion and proliferation. Furthermore, the presence of distributed bone matrix on all scaffolds was evaluated after 70 days as compared with PLLA only samples. Poly(hydroxyl butyrate)/chitosan (PHB/chitosan) blend scaffolds were prepared as a 3D subtract in cartilage tissue engineering by Kerbasi et al. [15]. It was revealed from this investigation that the crystallization of PHB in the blend was suppressed by increasing the chitosan content. The in vitro degradation rate of scaffolds was shown to be higher than the pure PHB scaffolds. A maximum degradation rate was observed for the scaffold containing 90% by weight. These authors suggested that these prepared scaffolds could serve as a three-dimensional substrate in cartilage tissue engineering.

Among the scaffolds made from polymer composites, Miao et al. [16] developed, using a combination of HA and TCP, hybrid materials characterized by high porosity (87%). The incorporation of PLGA in this composite significantly improved the compressive strength [17]. Kucharska et al. [18] described a technique to prepare biodegradable high compressive strength bone scaffolds. These authors used a method based on the agglomeration of composites of chitosan (CH) and chitosan/tricalcium phosphate (CH/TCP) microspheres to apply bone tissue engineering. The properties of these obtained material hybrids were significantly affected by the content of calcium phosphate, which had a particular influence on the granules’ microstructure, the size distribution, and the size of the internal pores of the biomaterial. The mechanical properties of the manufactured scaffolds had the potential for use as a load-bearing material. The number of cells was lower for the materials enriched in the inorganic phase, but the cellular distribution was recognized to be more even on the surface of the material containing the highest content of calcium phosphate.

Poly(δ-valerolactone) (PDVAL) belongs to the lactone family, which easily polymerizes by the ring-opening route [19] and can also be a potential candidate in biomedical applications. This polymer has a semi-crystalline aliphatic structure, with a low melting point and glass transition temperature, while exhibiting less elastomeric behavior than PCL [20]. PDVAL has good biodegradability, biocompatibility, and permeability characteristics making it a necessary and vital aliphatic polyester [21,22].

Poly(ethylene vinyl alcohol) (PEVAL) is a synthetic random copolymer of semi-crystalline structure over the entire range of composition [23]. This copolymer is synthesized by various processes, the most important of which is ethylene’s copolymerization with vinyl acetate, which produces poly(ethylene-co-vinylacetate) followed by hydrolysis of the vinyl alcohol units [24]. Being one of the most popular and flexible materials, PEVAL is largely investigated to be used in the biomedical field, notably as a carrier in the drug delivery field because of good blood compatibility [25], high gas-barrier [26], biocompatibility [25], harmlessness toward health [27], non-toxicity [28], hydrophilicity, availability of ethylene units to control its ability to swell and hence control the rate of its delivery [29], and has safe ethylene units liable to degradation and can undergo complete biodegradation, notably in the presence of enzymes [28,30].

Calcium phosphides (CaPs) are chemical compounds of special interest for human beings because of their similarity with the inorganic part of major normal calcified tissues of mammals such as bones, teeth, and antlers [31]. Its ability to promote bone healing was demonstrated a hundred years ago, and its osteoconductive properties and biocompatibility have been shown in experimental and clinical studies [32,33]. It has been recognized to be the most extensively incorporated natural or synthetic polymer to improve scaffold bioactivity [34]. Beta-tricalcium phosphate (β-TCP) is one of the most commonly used CaPs and has been shown to possess remarkable biocompatibility, osteoconductivity, bioresorbability, and direct bonding to bone experimentally [35] and clinically [36,37]. Moreover, it has been recently integrated with several polymers improving their characteristics as bone scaffolds [38,39,40].

One option to increase the surgeon’s operational freedom and resilience of ceramic-based bone graft replacement materials is to use composites to bond micro granular ceramic particles in a solid form with a biodegradable polymer blend matrix and to further form the hybrid material into a structure that mimics bone [41]. Therefore, synthetic biomaterials such as mixed polymer scaffolds have been developed but seldom studied [42,43,44,45] as an osteoconductive material combined with β-TCP particles in craniomaxillofacial surgery [46].

In this work, hybrid polymer material involved a PEVAL/PDVAL blend combined with β-TCP microparticles was selected as the composite for potential use in the biomedical field and more particularly for tissue engineering. To achieve this goal, the PEVAL/PDVAL/β-TCP composite containing 50 and 70 wt% of β-TCP content was prepared by the solvent casting method. The miscibility of the polymer blend and the dispersion of the β-TCP microparticles in the polymer matrix were examined by differential scanning calorimetry (DSC), X-ray diffraction (XRD), and scanning electron microscopy (SEM). Microcomputed tomographies (Micro-CT) were used to non-destructively and quantitatively measure three-dimensional percentage of porosity and pore size distribution in the blend and composites containing 50 and 70% by weight of β-TCP. The mechanical properties were studied through the tensile test analysis. Biological properties such as cell viability and proliferation were assessed using Alamar blue cell viability assay, and antibacterial activity was assessed using an inhibition test zone. To use these prepared hybrid materials as scaffolds releasing antibiotics, drug carrier systems containing PEVAL/PDVAL and PEVAL/PDVAL/β-TCP loaded with amoxicillin were chosen as the model prepared by the solvent casting route and a comparative in vitro study was carried out on this drug released in a medium of pH 7.4 [38,47].

## 2. Materials and Methods

### 2.1. Chemicals

PEVAL (purity 99.3%, ${\bar{M}}_{w}=2.0\times 10^{4}\;\mathrm{mol}\cdot g^{-1}$) containing 32 mol% ethylene, DVAL (purity 98%), N,N-dimethylformamide (DMF) (purity 97%), sodium chloride (purity 99.5%), and calcium phosphate powder (β-TCP) (phase purity ≥90%) were supplied from Sigma-Aldrich (Taufkirchen, Germany). Amoxicillin (purity ≥98%) (AMOX) was provided from Santa Cruz Biotechnology (Dallas, TX, USA). Solvents and precipitants such as chloroform (purity ~99.9%) and hexane (purity 99.5%), and isopropanol (purity 99.5%) were provided from BDH Prolabo. Alamar blue assay (AbD Serotec, Raleigh, NC, USA), mesenchymal stem cells (MSCs), and Dulbecco’s modified Eagle medium (DMEM) augmented with phosphate-buffered saline, and penicillin/streptomycin were supplied from (Invitrogen, CA, USA). All the chemicals were used without further purification.

### 2.2. Preparation of PDVAL/PEVAL/β-TCP Composite

PDVAL was synthesized using the procedure described in the literature [48]. Using a 50 mL flask, a predetermined amount of PDVAL/PEVAL (1:1 wt ratio) was completely dissolved in 10 mL of DMF at 80 °C under continuous stirring to prepare a polymeric solution. Then, a known quantity of β-TCP particles was added to the polymeric blend to form a suspension. The β-TCP particles suspended in the polymeric solution were dispersed by stirring using an ultra-sonic bath for 30 min to prevent aggregates’ formation. To remove any air bubbles that formed in the resulting film, the resulting PEVAL/PDVAL/β-TCP suspension was poured onto a Teflon plate, and then transferred to a desiccator maintained at reduced pressure, for 5 min. Finally, the plate containing the degassed composite mixture was allowed to dry at ambient temperature ~5 °C, for 48 h. To remove all solvent traces encrusted in the prepared composite, the whole, flat, and composite, was then transferred to a vacuum oven maintained at 50 °C until constant weight. A series of PEVAL/PDVAL/β-TCP was prepared using the same procedure. The prepared conditions are summarized in Table 1. Notably, a reference sample containing only the PEVAL/PDVAL blend was prepared under the same conditions mentioned to be used to compare analyses. 

### 2.3. Pores Interconnection

A defined amount of the PEVAL/PDVAL blend or the PEVAL/PDVAL/β-TCP composites was dissolved in a minimum of DMF at 80 °C with continuous stirring until complete dissolution. An equivalent amount of pure NaCl microparticles, having diameters varying between 200 and 400 μm used as a porogen, was added to the PEVAL/PDVAL solution or the PEVAL/PDVAL/β-TCP composites forming a very thick suspension. The resulting suspension was placed in an ultra-sonic bath for 10 min at 30 °C to ensure better dispersion and good distribution of the β-TCP particles and those of the porogen in the polymer matrix. Then, the suspension was poured into a steel mold giving the sample’s desired shape, then, left to dry at room temperature (25 °C) overnight, and then placed in vacuum desiccators for another 24 h at 40 °C for further removal of any solvent trace. The samples obtained were immersed in distilled water for 24 h to remove the porogen (particles of NaCl). To effectively remove the salt encrusted in the hybrid materials, the water was renewed every 4 h, and the samples were removed, dried, and weighed each time to constant weight. Then, the porous samples obtained were placed in a vacuum oven maintained at a temperature slightly lower than that of the hybrid material’s glass transition, where the connection of the pores was immediately made caused by the high-pressure difference exerted between the walls of the pores [46].

### 2.4. Characterization of PEVAL/PDVAL/β-TCP Composite

The PEVAL/PDVAL/β-TCP composites were characterized by different techniques. The DSC thermograms were obtained by a Shimadsu DSC 60 system (Japan) previously calibrated with indium. Samples weighing between 10–12 mg of polymer or composites were packed in aluminum DSC pans, before being placed in a DSC cell, and heated under nitrogen gas from 30 to 200 °C at a heating rate of 20 °C∙min^−1^. The data were collected from the second scan run for all samples. No degradation phenomena of polymer, copolymer, blends, and composites were observed in all DSC thermograms in the temperature range investigated. Notably, the T_g_ value was estimated as the midpoint of the heat capacity change with temperature, and the T_m_ at the top of the melting changes with temperature. XRD of pure β-TCP microparticles, polymer, copolymer, blend, and composites were recorded by a Rigaku D_max_ 2000 X-ray diffractometer using an anode tube of Cu working with a voltage of 40 KV and a generator current of 100 mA. The range of diffraction angle was 0–80 two theta. The samples were used as thin films except that of pure naphthalene, which was analyzed as powder.

The effect of incorporating β-TCP in the PEVAL/PDVAL matrix on its mechanical properties was studied using an Instron 5965 machine with Bluehill3 software (Instron, Elancourt, France). The crosshead speed of 5 kN load cell was set to 0.1 mm.min−1 until 15% deformation of the sample. The results of compressive strength were recorded (defined as compressive stress at maximum load). Five samples (*n* = 5) from each mixture were measured, before the test, using a digital clipper (500, Mitutoyo, CA, USA).

Surface morphology analyses of dried samples were performed on a Jeol JSM-6360LV SEM (Tokyo, Japan) at an accelerating voltage of 10 kV. The surface and cross-sections of samples were first sputter-coated with a thin layer of gold, and then observed at a magnification range of 300–3000×.

To non-destructively and quantitatively measure three-dimensional (3D) porosity percentage and pore size distribution among the PEVAL/PDVAL blend, PEVAL/PDVAL/β-TCP50, and PEVAL/PDVAL/β-TCP70, micro computed tomography (micro-CT) were used. Five cylindrical samples measuring 6 × 6 mm from each group were scanned with a SkyScan 1172 micro-CT imaging system (Bruker SkyScan, Kontich, Belgium) at 10 mm resolution using a voltage of 52 kV, and a current of 188 mA, 885 ms exposure time, and isotropic resolution of 9.85 µm image pixel size. The volume of interest with 1 mm less than the sample size was selected in the center of a scaffold to eliminate potential edge effects. The volume of internal porosities was quantified with the CTAn^®^ program version 1.17.7.2 (Bruker Skyscan, Kontich, Belgium) by image segmentation.

### 2.5. In Vitro Biocompatibility

Cell viability on the hMSCs was done on Days 1, 2, and 3 to assess the in vitro biocompatibility of the PEVAL/PDVAL blend, and the PEVAL/PDVAL/β-TCP50 and PEVAL/PDVAL/β-TCP70 preparations were compared to cells grown on a culture plate well as positive control.

#### 2.5.1. Cell Culture

Human mesenchymal stem cells (hMSCs), a sub-clone (CL1) derived from hMSC-TERT, were used. These cells have been comprehensively studied and characterized to show similarities to hMSCs in cellular and molecular phenotypes (Elsafadi et al., 2016). They also exhibited greater differentiation capacity as they were found to form mineralized nodules at 7 days of differentiation. The cells were cultured in DMEM supplemented with 0.25 mol/L D-glucose, 0.004 mol/L L-glutamine, 0.006 mol/L sodium pyruvate, 10% fetal bovine serum (FBS), 1× penicillin-streptomycin (Pen-strep), and non-essential amino acids.

#### 2.5.2. Cell Seeding and Viability Assessment

Disks from each preparation were placed in 24-well plates and immersed with DMEM culture medium supplemented with the same materials mentioned earlier and kept overnight at 37 °C. Then, cells were seeded into the wells (containing disks from different scaffold preparations and empty wells with culture medium only as control) at a seeding density of 0.08 × 10^6^ cells/well and cultured at 37 °C in an incubator with a humidified atmosphere of 5% CO_2_ for 3 days. Assessment of cell viability was done in triplicate, based on the International standard ISO 10993-5 concerning tests for in vitro cytotoxicity of medical devices (indicating a minimum number of repeats of *n* = 3, as used in this experiment). Samples resulting in cell viability above 70% of the control were considered to be non-cytotoxic. Alamar blue assay was utilized, according to the manufacturer’s recommendations (AbD Serotec, Raleigh, NC, USA). Briefly, 10 μL of Alamar blue substrate was added, and the plates were incubated in the dark at 37 °C for 1 h. A reading was subsequently taken by measuring the cells’ fluorescence (Ex 530 nm/Em 590 nm) with a BioTek Synergy II microplate reader (BioTek Inc., Winooski, VT, USA) [49,50,51].

The zone of inhibition test was conducted to evaluate the effect of adding the antibiotic to the PEVAL/PDVAL blend, and the PEVAL/PDVAL/β-TCP50 and PEVAL/PDVAL/β-TCP70 preparations. A bacterial broth of Staphylococcus aureus bacterial strain (ATCC 29213) was prepared in 5 mL of brain heart infusion and incubated at 37 °C for 24 h. Ten μL of the bacterial suspension were spread on a blood-agar plate. The plates were incubated for 24 h to permit bacterial colonies to grow. Subsequently, a few bacterial colonies were dissolved in a container with physiological solution, a 0.5 McFarland standards turbidity solution (approximately (1–2) × 10^8^ CFU∙mL^−1^) was prepared (turbidity was assessed using a BD PhoenixSpec™ nephelometer, Franklin Lakes, NJ, USA). Afterwards, a sample of the suspension was spread over brain heart infusion agar plates.

Drug incorporation was done by adding 500 mg amoxicillin to 1.0 g of the PEVAL/PDVAL blend, and the PEVAL/PDVAL/β-TCP50 and PEVAL/PDVAL/β-TCP70 DMF mixtures at 50 °C under continuous stirring until complete dissolution, thus, forming a thick viscous solution, which was poured onto a custom-designed mold over a horizontal plate and left to dry at ambient temperature for 48 h, followed by drying in a vacuum oven at 40 °C. Subsequently, films were placed on the bacterial colonies in the brain heart infusion plate, to be incubated at 37 °C for 24 h. Samples of each mixture were incubated for 24 h at 37 °C in the brain heart infusion agar plates, and the zone of inhibition represented by the halo formed around the sample was measured. Tests were done in triplicate, respectively.

### 2.6. In Vitro Drug Release Studies

The PEVAL/PDVAL/β-TCP/AMOX composite films were suspended in 100 mL of aqueous solution fixed at pH media of 7.4 and stirred at 100 rpm at 37 °C (body temperature). Aliquots of 0.5 mL were taken at time intervals for analysis, and then immediately replaced by an equivalent volume of the initial solution, taking into account in the calculations the amount of drug in the sample rejected. This comes closer to what occurs during the release of the drug in real time, the released quantity of which is immediately absorbed by the organs. This operation kept a constant volume of media during the release process. As mentioned above, the total mass of AMOX released during a certain duration was calculated from the absorbance determined. It is important to note that the pH of water was practically not affected by the small amount of drug released during the release process. Indeed, the quantity of amoxicillin released was negligible as compared with that of the medium, and, in addition, this drug has an amphoteric character with a pKa of 6.93 [52,53] close to the pH of the release medium. This indicated that the addition of a buffer solution to the medium was not necessary.

## 3. Results and Discussion

### 3.1. Characterization

#### 3.1.1. Differential Scanning Calorimetry (DSC) Analysis

The miscibility of the PEVAL/PDVAL system has already been proven in our previous work [46] by DSC through the presence of an ultimate glass transition temperature for each composition investigated and the negative value of the Flory interaction parameter and confirmed by the presence of hydrogen bond between the carbonyl groups of the DVAL units and the hydroxyl groups of the EVAL units.

**Figure 1:**
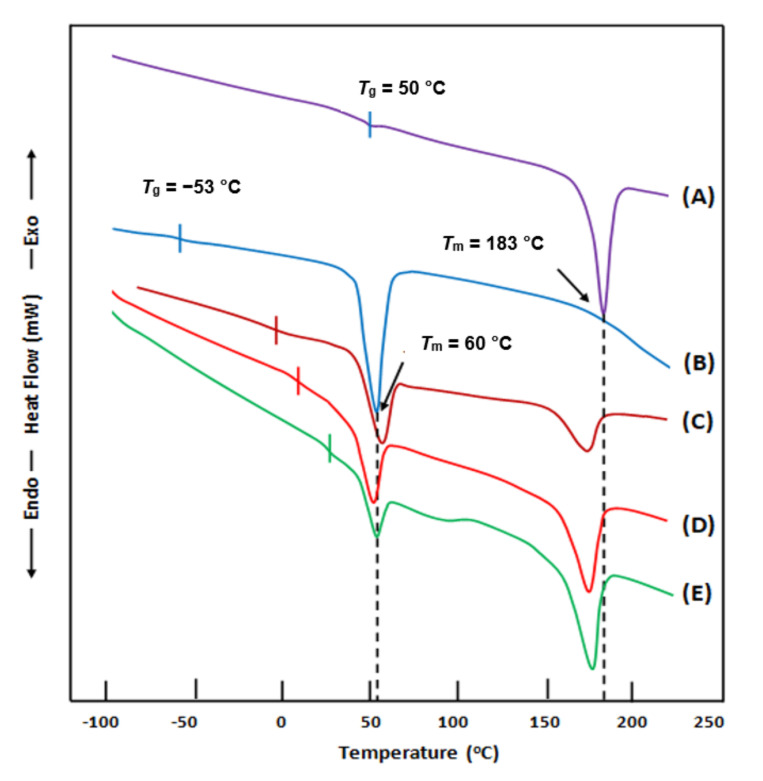
Differential scanning calorimetry (DSC) thermograms of (**A**) poly(δ-valerolactone) (PEVAL); (**B**) poly(ethylene-co-vinylalcohol) (PDVAL); (**C**) PEVAL/PDVAL blend; (**D**) PEVAL/PDVAL/β-TCP70 composite; (**E**) PEVAL/PDVAL/β-TCP50 composite. Here, β-TCP70 and β-TCP50 represent 70 and 50 wt% of beta-tricalcium phosphate.

The effect of the incorporation of β-TCP microparticles in the PEVAL/PDVAL matrix on the blend’s thermal behavior is shown on the DSC thermograms, in Figure 1. As can be seen from these thermal curves, the important shift in the glass transition temperature of the blend to the right indicates strong adhesive forces of the filler on the polymer chains. Indeed, the presence of strong interactions between the polymer chains and the β-TCP microparticles prevents the chains from sliding between them. In this case, overcoming these interaction forces and facilitating the chains’ movement requires the absorption of more heating energy. Through these thermograms, we can also observe a shift toward the low temperatures in the melting temperature of PEVAL in the blend in the composites. This shift is because PEVAL, which has a much higher melting temperature than PDVAL, always remains in its solid state, while the latter is already in its melting state. Under these conditions, there is a phenomenon of softening of the PEVAL chains in the mixture, resembling a partial dissolution of this copolymer in the PDVAL in the mixture leading to its early melting. This study revealed that the β-TCP microparticles were dispersed uniformly in the PEVAL/PDVAL matrix, which was proven by the change in the thermal behavior of the resulted hybrid materials.

#### 3.1.2. X-ray Diffraction (XRD) Analysis

**Figure 2:**
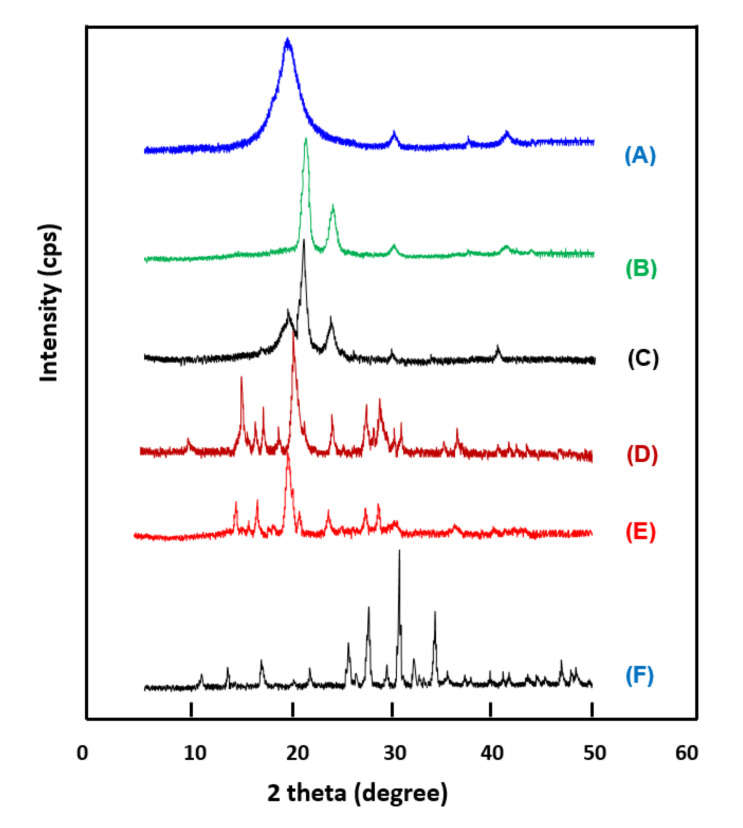
X-ray diffraction (XRD) patterns. (**A**) PEVAL; (**B**) PDVAL; (**C**) PEVAL/PDVAL; (**D**) PEVAL/PDVAL/β-TCP50; (**E**) PEVAL/PDVAL/β-TCP70; (**F**) pure β-TCP.

The XRD patterns of the PEVAL/PDVAL/β-TCP composites and their pure components are shown in Figure 2. Beta-TCP confirms its crystalline structure by the presence of four principal reflection signals centered at 26.2, 27.8, 31.2, and 34.2°, which agree with those of the literature [54,55]. The PEVAL/PDVAL blend brings together, practically, the characteristic peaks of each component, in which the spectrum of the copolymer exhibits its broad signal at 20.0° [56] and that of the polymer shows its two main peaks, one of which is intense at 22.1° and the other less intense at 24.5° [57]. The absence of change in the relief of the spectrum of the mixture as compared with that of the two components indicates that PEVAL and PDVAL retain their orthorhombic crystalline structures in the blend in two distinct crystalline regions, i.e., one rich in PEVAL and the other rich in PDVAL. For the hybrid material PEVAL/PDVAL/β-TCP, the total disappearance of the two main signals of the four at 26.2 and 34.2°, and the simultaneous appearance of two other peaks at 15 and 19°, indicate a modification of the crystal structure of β-TCP in composites. A significant depression of the intense signal at 22.1° belonging to the PDVAL is observed, which also reveals a modification of the crystal structure of this polymer in the mixture. Consequently, we can conclude that the incorporation of β-TCP particles into the mixture had a reciprocal effect with the PDVAL component; in addition, the structure of PEVAL remained intact.

#### 3.1.3. Scanning Electron Microscopy (SEM) Analysis

**Figure 3:**
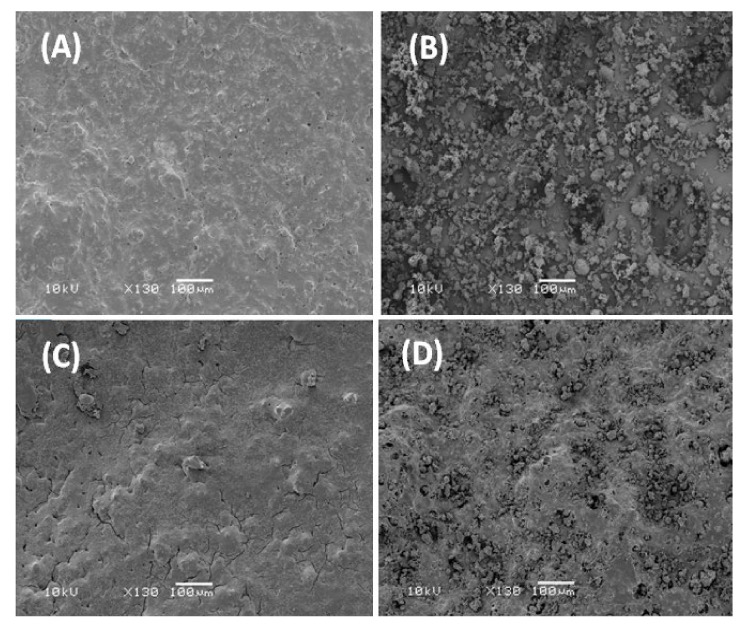
Scanning electron microscopy (SEM) images (**A**) PEVAL/PDVAL blend; (**B)** β-TCP microparticles, (**C**) PEVAL/PDVAL/β-TCP50; (**D**) PEVAL/PDVAL/β-TCP70 composites zoomed at 130×.

Figure 3 shows the micrographs of β-TCP microparticles, surface morphologies of the PEVAL/PDVAL and PEVAL/PDVAL/β-TCP samples containing 50 and 70 wt% of β-TCP fillers, respectively, used as reference taken before pores connect. As shown in the β-TCP image, the particles do not have particular shapes where they seem to be gathered in aggregates of average diameter size varying between 10 and 35 μm.

Concerning the PEVAL/PDVAL blend, the corresponding image shows a homogeneous, slightly grainy surface devoid of any heterogeneous zone, thus, confirming this system’s miscibility. The photos in the bottom show the surface morphology of the PEVAL/PDVAL/β-TCP50 and PEVAL/PDVAL/β-TCP70 hybrid materials and reveal, for the first sample, a total coverage of the filler particles distributed uniformly in the polymeric matrix resembling torrential mud deposited on stony ground, while in the second photo, an excess of β-TCP particles remains practically uncovered with the polymer.

**Figure 4:**
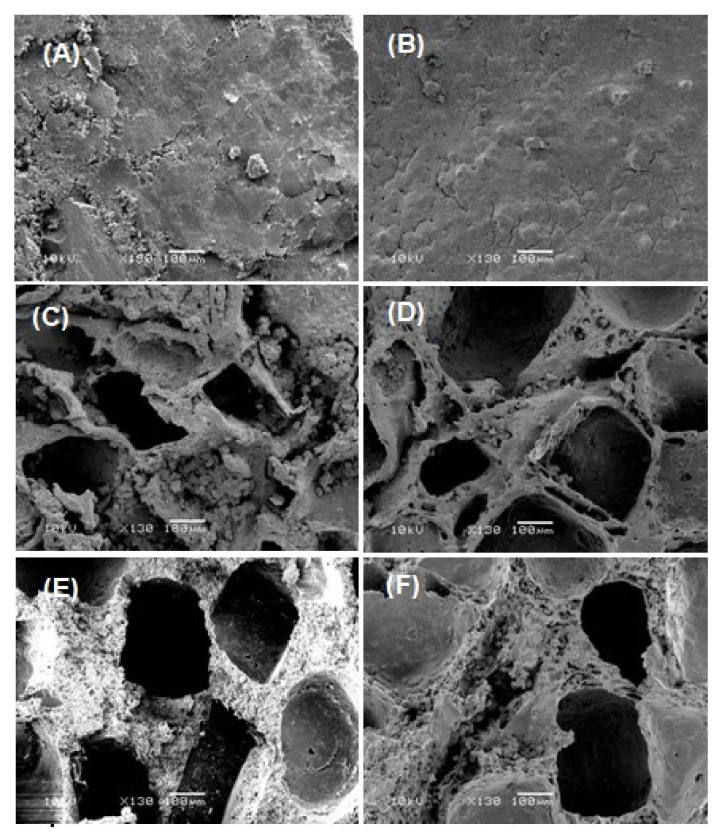
SEM images of surface morphologies of: (**A**) PEVAL/PDVAL/β-TCP70 (before pore connection); (**B**) PEVAL/PDVAL/β-TCP50 (before pore connection); (**C**) PEVAL/PDVAL/β-TCP70 (after pore connection); (**D**) PEVAL/PDVAL/β-TCP50 (after pore connection) and cross section morphologies of: (**E**) PEVAL/PDVAL/β-TCP70 (after pore connection); (**F**) PEVAL/PDVAL/β-TCP50 (after pore connection) zoomed at 130×.

The images in Figure 4 show the samples of the PEVAL/PDVAL/β-TCP50 and PEVAL/PDVAL/β-TCP70 hybrid materials after the formation of the interconnected pores. As can be seen from these micrographs taken from the surface and the cross-section of the samples, the scaffolds both have interconnected pores of oval or cubic shape of average size ranging between 300 × 450 μm and 200 × 250 μm, which perfectly reflect the particle size of the salt used as porogen. A comparison of the two scaffolds reveals that the pores’ density is not affected by the β-TCP amount in the polymer blend. Both hybrid materials’ porosity responds well to the conditions required to allow their uses as scaffolds in the biomedical field [58,59]. Indeed, general porosity, pore size, and pore interconnectivity are three parameters required to meet a candidate material’s cellular conditions for its use as a scaffold [60,61]. According to different authors [58,59], the appropriate pore sizes for bone formation purposes are greater than 300 µm, to allow sufficient vascularization of the material and to avoid hypoxic conditions in internal regions [58,59]. This is consistent with our observation using non-cross polymerized poly (lactic acid-co-glycolic acid) scaffolds [60]. In this case, pore sizes ranging from 300 to 500 μm gave the best performance for collagen production, hydroxyapatite deposition, and bone mineral maturation.

#### 3.1.4. Porosity

**Figure 5:**
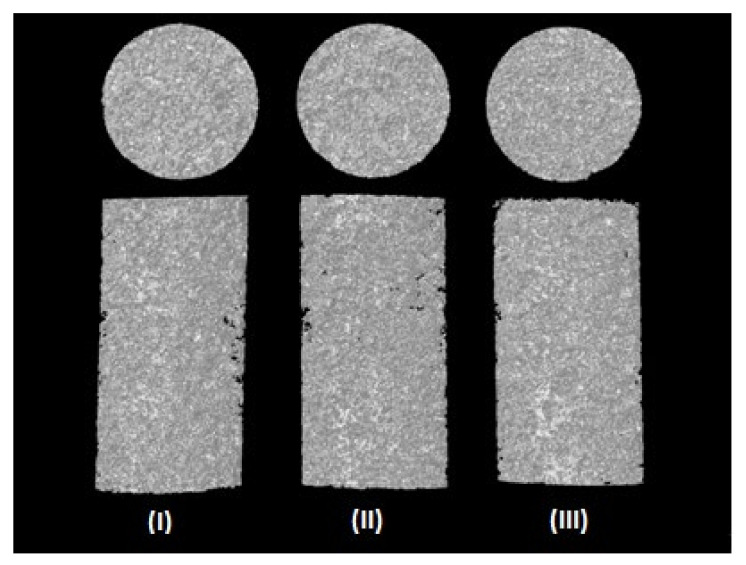
Representative three-dimensional (3D) images of the porous PEVAL/PDVAL blend (**I**), and the PEVAL/PDVAL/β-TCP50 (**II**) and PEVAL/PDVAL/β-TCP70 (**III**) hybrid materials.

**Figure 6:**
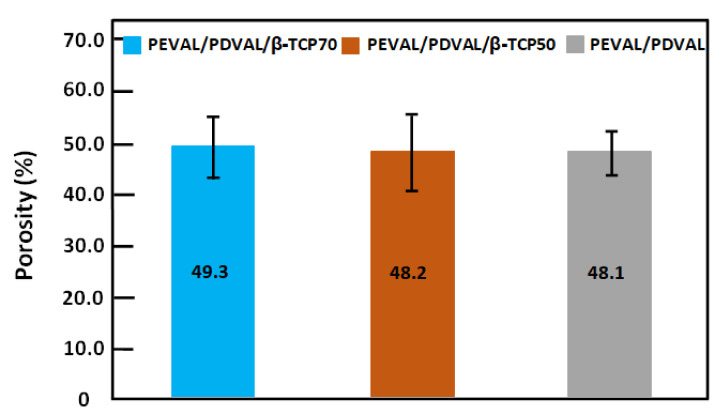
Results of micro-CT analysis. Porosity of the PEVAL/PDVAL blend, and the PEVAL/PDVAL/β-TCP50 and PEVAL/PDVAL/β-TCP70 hybrid materials, as determined by micro-CT. Error bars represent standard deviations, *n* = 5.

Representative 3D images of the porous PEVAL/PDVAL blend (I), and the PEVAL/PDVAL/β-TCP50 (II) and PEVAL/PDVAL/β-TCP70 (III) hybrid materials reconstructed by micro-CT are presented in Figure 5. The porosity of each specimen after salt leaching was determined from micro-CT data, and are shown in Figure 6. The resulted porosity percentage came consistently lower but in close coordination to the planned one (50% by weight). The results showed no significant difference in the porosity percentage among the three scaffolds, in which the porosity for the PEVAL/PDVAL blend was 48 ± 4.2%, that of PEVAL/PDVAL/β-TCP50 was 48.2 ± 7.2%, and that of PEVAL/PDVAL/β-TCP70 was 49.2 ± 5.9%.

**Figure 7:**
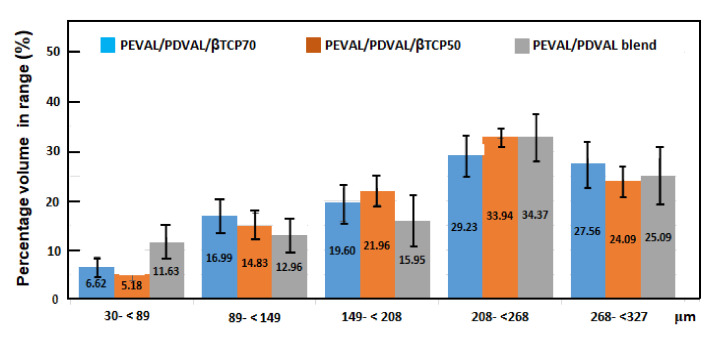
Percentage of pore size ranges among the PEVAL/PDVAL blend, and the PEVAL/PDVAL/β-TCP50 and PEVAL/PDVAL/β-TCP70 hybrid materials, as determined by micro-CT.

Regarding the pore size frequency of the scanned samples, presented in Figure 7, the same pore size distribution pattern was observed in the three investigated polymeric materials. The peak pore size, in all samples, ranged from 208 to less than 268 μm with 29.2 ± 4.5%, 33.9 ± 1.4%, and 34.3 ± 14.9% for the PEVAL/PDVAL/β-TCP70, the PEVAL/PDVAL/β-TCP50 hybrid materials, and the PEVAL/PDVAL blend, respectively. The second most predominant pore size ranged from 268 to less than 327 μm, followed by the pore size of 149 to less than 208 μm. The least frequent pore size was the pore size ranging from 30 to less than 89 μm, in which the PEVAL/PDVAL/β-TCP70 and PEVAL/PDVAL/β-TCP50 hybrid materials had 6.62 ± 4.1% and 5.18 ± 3.6%, respectively, which was significantly lower than the PEVAL/PDVAL blend with 11.63 ± 13.1%.

The present porosity in any scaffold planned for bone regeneration is one of the main requirements to simulate natural bone morphology. Different methods and porogen types have been used for scaffold pore formation [58]. However, NaCl microparticle leaching was used in this study as it has been proven in the literature to provide a controlled pore size ranging from macroporous to microporous size [62]. A pore size ranging from 200–400 µm has been suggested to be optimal due to the similarity with human osteon size 223 µm [63]. In the present study, the resulting porosity percentage was concordant with the intended porosity percentage. The none significant decrease presented in the PEVAL/PDVAL blend group as compared with the hybrid materials was in contrast to the result of Mao et al. [64], in which the porosity percentage decreased with an increase in HA concentration to the polymer blend. However, the porosity percentage in this study (approximately 49%) was in agreement with Shi et al. [65], as it was slightly lower than the intended percentage (50%).

#### 3.1.5. Mechanical Testing

**Figure 8:**
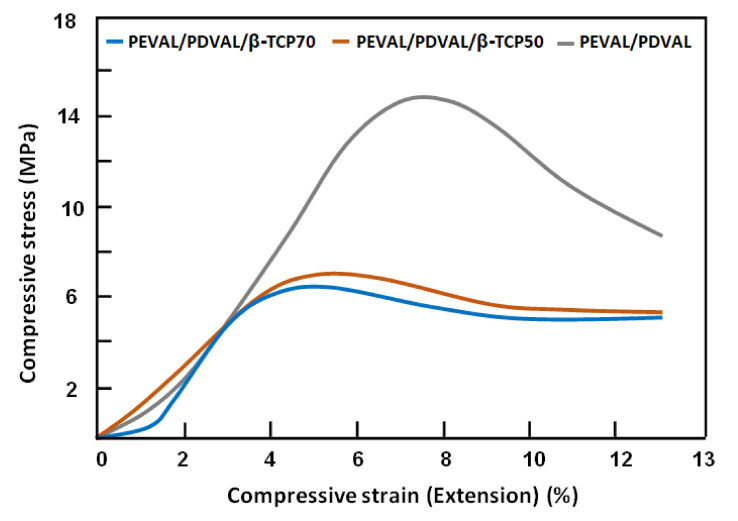
The stress-strain curves resulted from the compressive tests of the PEVAL/PDVAL/β-TCP specimens with different β-TCP content and that of the virgin blend.

An important outcome of any bone substitute is the mechanical properties. The stress-strain curve resulting from the compressive test of each mixture is shown in Figure 8. Compressive strength significantly decreased as β-TCP particles were added to the PEVAL/PDVAL blend (15 ± 2.7 MPa) *p* < 0.05. However, the compressive strength did not significantly differ between PEVAL/PDVAL/β-TCP70 and PEVAL/PDVAL/β-TCP50 by 6.3 ± 1.3 MPa and 7.0 ± 1.4 MPa, respectively. The compressive modulus showed a similar pattern of difference, in that, the β-TCP addition significantly decreased the compressive modulus for PEVAL/PDVAL/β-TCP70 (51.6 ± 8.4 MPa) and PEVAL/PDVAL/β-TCP50 (59.9 ± 7.5 MPa) as compared with the compressive modulus for the PEVAL/PDVAL blend of 129 ± 6.9 MPa.

Mechanical properties of β-TCP and other CaPs ceramics can be improved by adding different polymers [66]. In this study, the addition of the PEVAL/PDVAL blend increased the compressive strength concerning the percentage of polymer added (6.3 ± 1.3 MPa and 7.0 ± 1.4 MPa for 30% and 50% of the PEVAL/PDVAL blend percentage). These results agreed with Kang et al. [67], in which the compressive strength of β-TCP scaffolds significantly improved from 2.90 to 4.19 MPa after the infusion of PLGA. Additionally, Kim et al. [68] found that coating hydroxyapatite with poly(ε-caprolactone) exhibited higher compressive strengths. In the coated specimens, the higher concentration showed a higher strength. Our results showed that incorporating β-TCP with the PEVAL/PDVAL blend resulted in a compressive strength similar to human cancellous bone (2–10 MPa) [69].

#### 3.1.6. Cellular Viability and Proliferation

**Figure 9:**
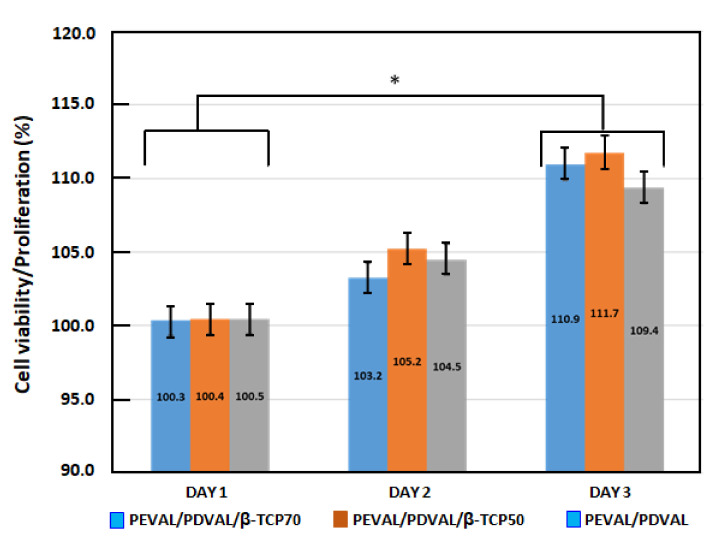
Cell viability and proliferation results on PEVAL/PDVAL/β-TCP hybrid material scaffolds containing 50 and 70 wt% of β-TCP contents and the PEVAL/PDVAL blend (reference during 1, 2, and 3 days). * Statistically significant difference (*p*-value < 0.05).

The results of hMSC proliferation tests obtained are shown in Figure 9. Alamar blue assay showed that hMSCs could grow and proliferate on the different types of the prepared scaffolds, reflecting that the hybrid materials are non-toxic and can be biologically acceptable as scaffolds. Cell proliferation was found to be increased over time for the PEVAL/PDVAL blend and incorporated TCP particles, in which there was a significant increase in cellular proliferation between Day 1 and Day 3. However, no statistically significant difference was observed between the groups or between Day 2 and Day 1 or Day 3. This is quite clear for the PEVAL/PDVAL/β-TCP50 composite, which showed a steady increase in cell proliferation from Day 1. Although cell proliferation in the PEVAL/PDVAL/β-TCP70 composite was slightly lower than in the PEVAL/PDVAL blend on Day 2, it showed significant results after Day 2. This agrees with that of the literature [3], in which higher cell proliferation in three days was associated with a higher β-TCP concentration in the hybrid material.

The improvement in the adhesion capacity of the cells on the PEVAL/PDVAL mixture is probably due to the following two essential factors: an increase in the physico-chemical affinity due to the hydrogen bonds developed between the cells and the hydroxyl groups of the vinyl units in PEVAL and an increase in the density of the pores at the surface of the material obtained when the hydroxyl groups carried by PEVAL were added to PDVAL. When the content of PEVAL in the blend was more than 50% by weight, the decrease in the adhesion performance was due to the reduction in the size of the pores on the surface of the resulting material caused by the excessive attraction forces created by increasing the density of the hydroxyls of the polymeric blend. Similar results were also obtained by Alghamdi et al. [70] in an investigation that involved PEVAL and PCL.

#### 3.1.7. Antibacterial Activity

**Figure 10:**
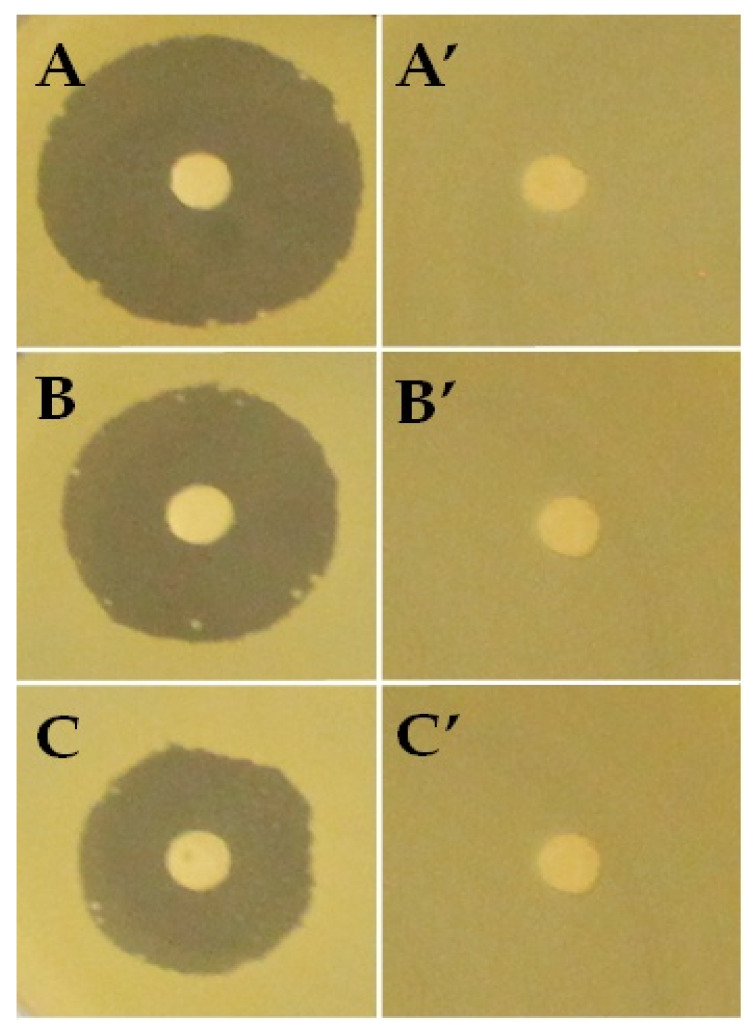
Antibacterial activity. (**A**) PEVAL/PDVAL/β-TCP70; (**B**) PEVAL/PDVAL/β-TCP50; (**C**) PEVAL/PDVAL scaffolds with antibiotic and (**A’**); (**B’**,**C’**) without antibiotic.

Antibacterial activity is a fundamental factor in bone scaffolding’s success, as an infection caused by bacterial invasion of a surgically created wound in general and bone, in particular, poses a major problem in healing. Therefore, the composite scaffold’s ability to be loaded with an antibiotic was assessed via using a zone of inhibition test. The incorporation of amoxicillin showed its ability to inhibit the bacterial growth of *S. aureus* in the brain heart infusion agar plates, 24 h after setting up the scaffold. Indeed, Figure 10 shows inhibition zones measuring 23.4, 20.6, and 18.3 mm in diameter for PEVAL/PDVAL/β-TCP70, PEVAL/PDVAL/β-TCP50, and PEVAL/PDVAL scaffolds, respectively, revealing that the higher the β-TCP concentration in the scaffold, the larger the diameter of the zone of bacterial inhibition.

#### 3.1.8. Statistical Analysis

Results of material’s porosity, cellular viability, and mechanical compression were statistically analyzed using SPSS, version 13. After confirming the normality of the variance’s distribution and homogeneity, the effect between the PEVAL/PDVAL blend, PEVAL/PDVAL/β-TCP50, and PEVAL/PDVAL/β-TCP70 was compared using one-way ANOVA. The Tukey post hoc test was used to detect the significant difference at the level *p* < 0.05 among the three groups. Data are reported as mean, standard deviation values.

### 3.2. Kinetics Release of Amoxicillin

**Figure 11:**
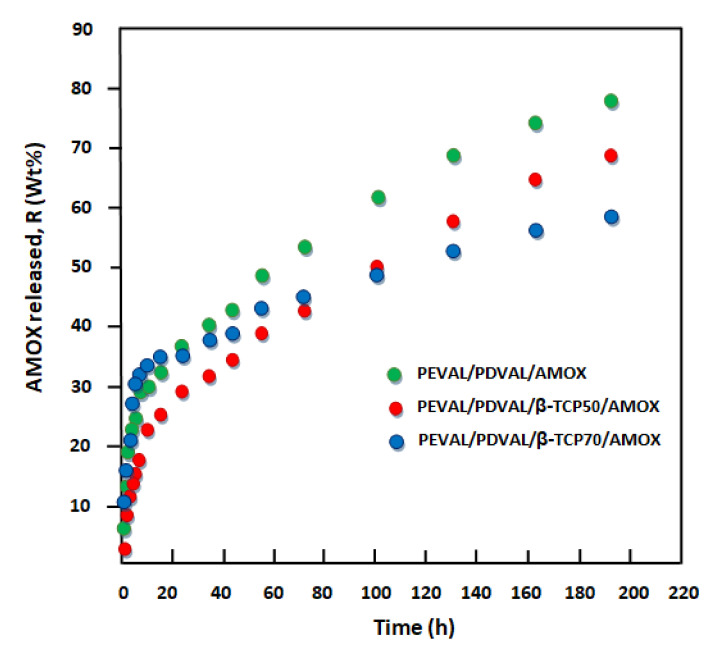
Cumulative amoxicillin released from the PEVAL/PDVAL/β-TCP/AMOX drug carrier systems.

The cumulative amoxicillin released, *R*, from PEVAL/PDVAL and PEVAL/PDVAL/β-TCP/AMOX drug carrier systems during eight days of the pH release process, media 7.4 and at 37 °C, was determined using Equation (1) as follows (the results obtained are plotted in Figure 11):

(1) $$R\left(\mathrm{wt}\%\right)=\frac{m_{t}}{m_{o}}\times 100$$

where *m_o_* and *m_t_* are the initial mass of amoxicillin in the drug carrier system, and that released at *t* time of the release process, respectively. As shown from these curve profiles, the percentage of amoxicillin released versus time followed a pseudo logarithmic growth for all specimens, in which the maximum percentage of drug released during this period varied between 78 wt% reached with the blend system and 58 wt% with the composite containing 70 wt% β-TCP of the total drug amount loaded initially. This revealed that the incorporation of β-TCP microparticles in the blend matrix considerably reduced the release dynamic of AMOX, and the percentage release of this drug decreased when the β-TCP content in the composite increased. The curve profiles also indicate that AMOX release dynamics during this process followed two principal distinct steps. The first step was rapid and short and occurred at about 5 h into the release process, in which between 22.65 and 33.70 wt% of the drug was consistently released during this period. The second step was much longer (151–153 h) and slower, in which between 25 and 48 wt% of AMOX was released depending on the percentage of β-TCP in the polymer matrix. Such behavior, usually observed by different authors in the drug delivery domain [71], can probably be attributed to both sides of the specimen’s leaching. A large number of drug particles attach or lightly embed on the surface and are immediately dissolved in the media. Next, comes the release of the rest of the drug particles embedded in the polymer matrix after dissolving by the penetrating media. During this period, the release of AMOX takes place in a long step characterized by a mechanism based on the dissolution–diffusion–desorption phenomenon. The release rate of AMOX released during each step was taken from the slope of the linear portion of the release curves of Figure 11, and the results obtained are grouped with those of the maximum drug released during these different periods in Table 2.

**Figure 12:**
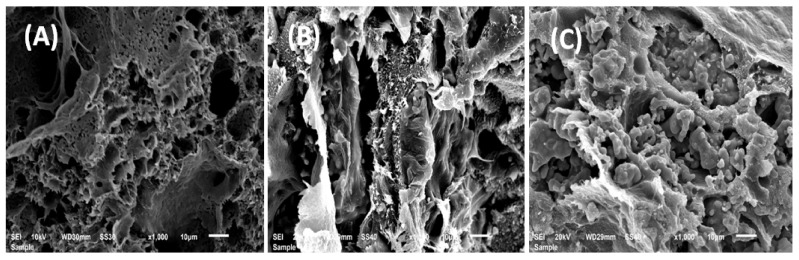
SEM images of the cross-section of (**A**) the PEVAL/PDVAL blend, (**B**) the PEVAL/PDVAL/β-TCP50 and (**C**) PEVAL/PDVAL/β-TCP70 composite specimens, zoomed at 1000×.

From these curve profiles, except for the virgin PEVAL/PDVAL system, it was also revealed that the best performance, leading to a maximum amount of released amoxicillin was obtained with the system containing 50% by weight of β-TCP, in which a total of 64.65 wt% of this antibiotic was released over eight days as compared with that which contained 70% by weight, in which 58.70 wt% was released during the same period. The reduction of the release dynamic observed when the β-TCP was incorporated in the virgin PEVAL/PDVAL blend could be explained through the photos obtained by SEM of cross-sections of samples before and after incorporating this filler, as shown in Figure 12. As can be seen from these images, the virgin polymer blend (top image) shows a large pore density of variable size dispersed throughout the polymer matrix. Two other images (in the middle and bottom), attributed to the composites, show that a large number of these pores were filled with β-TCP particles. Under these conditions, the amount of liquid entering these pores is reduced; therefore, the amount of AMOX dissolved in the media within the polymer matrix is also reduced. The incorporation of β-TCP particles considerably reduces the large pore density considered to be a reservoir for storing the media solution inside the virgin material. As the quantity of filler increases in the polymeric material, the density of the pores decreases. This is in the sense of reducing the amount of AMOX dissolved inside the material, causing a decrease in the amount of drug released.

#### Diffusion Behavior of Amoxicillin

**Figure 13:**
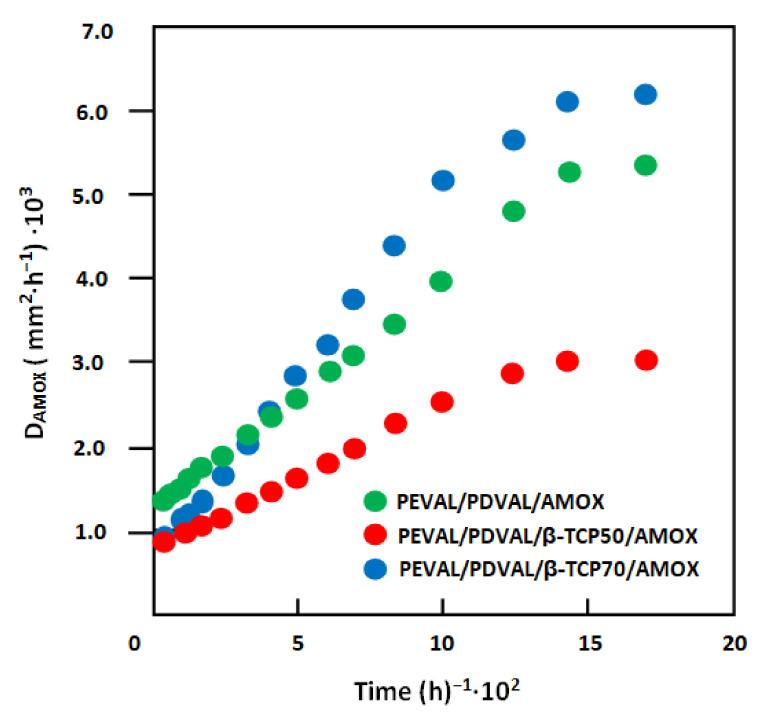
Variation of amoxicillin’s diffusion coefficient through the PEVAL/PDVAL/β-TCP/amoxicillin drug carrier systems with different β-TCP contents versus the inverse of time (revoir β-TCP).

According to Lin et al. [72], the diffusion of a drug through a polymeric material obeys the Fickian model if the cumulative drug released in a medium does not exceed 60 wt% of the total drug amount loaded in the polymer matrix. In this condition, the value of amoxicillin’s diffusion coefficient in the PEVAL/PDVAL/β-TCP is calculated from Equation (2) [73] as:

(2) $$D_{AMOX}=\frac{0.196\times l^{2}}{t}\times {\left[\frac{m_{t}}{m_{o}}\right]}^{2}$$

where *l* is the thickness of the specimen. *D_AMOX_* value is determined when the permanent regime is reached; therefore, all amoxicillin particles deposited or embedded on the film surface are leached by washing in the water media. In these conditions, the curve profiles of *D_AMOX_* versus time are meaningful and reflect exactly the dynamics of this medication released in the media inside the polymer matrix. For the PEVAL/PDVAL/AMOX and PEVAL/PDVAL/β-TCP/AMOX drug carrier systems, the diffusion coefficient’s variation versus the inverse of time calculated from Equation (2) and the experimental data of Figure 12 are gathered in Figure 13. As shown from these curve profiles obtained, straight lines were obtained for each drug carrier system. This finding indicates that amoxicillin’s diffusion through the PEVAL/PDVAL/β-TCP carrier effectively obeys the Fickian model. This finding also indicates that the permanent regime of the dynamic release was reached. Due to these results, it was possible to build our investigation on the second zone of the release process, which was generally localized between 5 and 192 h.

## 4. Conclusions

A new type of hybrid material involving the PEVAL/PDVAL miscible blend and β-TCP were successfully prepared by solvent casting route. The DSC and XRD methods analyses revealed that the blend was miscible, and the β-TCP microparticles were homogeneously distributed in the polymer blend matrix. The XRD analysis of the prepared hybrid materials indicates that the incorporation of β-TCP microparticles in the PEVAL/PDVAL blend affected both the crystalline structure of β-TCP and that of PDVAL in the composite. The SEM micrographs, taken from the surface and cross-section of the blend and hybrid materials after salt leaching, showed interconnected micropores that responded to the conditions required to allow their uses as scaffolds in the biomedical field. After salt leaching, the porosity of each specimen, determined from micro-CT data, indicated that the resulting porosity percentage was consistently lower but in close coordination to the planned porosity (50% by weight). No significant difference was revealed in the porosity percentage among the three scaffolds, in which the porosity for blend and composites varied between 48 and 49.2%. The stress-strain curve resulting from these specimens’ compressive tests revealed that the compressive strength significantly decreased as β-TCP microparticles incorporated in the PEVAL/PDVAL blend increased. The incorporation of β-TCP microparticles into the PEVAL/PDVAL mixture’s matrix improved the compressive strength of the blend similar to that of human cancellous bone (2–10 MPa). The proliferation test results revealed that the hMSCs could grow and proliferate on the different types of prepared materials, thus, reflecting that the hybrid materials were non-toxic and may be biologically acceptable as scaffolds. The antibacterial activity test revealed that the incorporation of amoxicillin in the specimens showed its ability to inhibit *S. aureus*’ bacterial growth of *S. aureus* and revealed that the higher β-TCP concentration in the scaffold, the larger the diameter of the bacterial inhibition zone. The in vitro amoxicillin released from the blend and hybrid materials revealed a maximum release of 78 wt% with the virgin blend during eight days. The incorporation of β-TCP microparticles in this blend revealed a significant reduction of amoxicillin’s release dynamic in the media pH 7.4. The mass transfer study results revealed that the diffusion of amoxicillin in the blend and composites scaffolds obeyed a Fickian model.

## Figures and Tables

**Table 1:** Preparation conditions of the poly(δ-valerolactone)/poly(ethylene-co-vinylalcohol)/β-tricalcium phosphate (PEVAL/PDVAL/β-TCP) composites.

System	PEVAL (g)	PDVAL (g)	β-TCP (g)	β-TCP (wt%)
PEVAL/PDVAL0	0.50	0.50	0	0
PEVAL/PDVAL/β-TCP50	0.25	0.25	0.5	50
PEVAL/PDVAL/β-TCP70	0.15	0.15	0.7	70

**Table 2:** Stable zones and instantaneous release of amoxicillin from PEVAL/PDVAL/AMOX and PEVAL/PDVAL/β-TCP/AMOX drug carrier systems with different β-TCP compositions.

Drug Carrier System	Stable Zone (h)	Amoxicillin Released (wt%)	Release Rate (wt%∙h^−1^)
PEVAL/PDVAL/AMOX	0–5	30.10 ± 0.34	6.02 ± 0.02
	5–158	48.02 ± 3.03	0.31 ± 0.06
PEVAL/PDVAL/β-TCP50/AMOX	0–5	22.65 ± 0.35	4.53 ± 0.03
	5–156	42.0 ± 2.50	0.27 ± 0.05
PEVAL/PDVAL/β-TCP70/AMOX	0–5	33.70 ± 2.50	6.73 ± 0.02
	5–156	25.0 ± 0.42	0.16 ± 0.02

## Data Availability

Data sharing not applicable.
